# Characterization of Rad51 from Apicomplexan Parasite *Toxoplasma gondii*: An Implication for Inefficient Gene Targeting

**DOI:** 10.1371/journal.pone.0041925

**Published:** 2012-07-30

**Authors:** Sita Swati Achanta, Shalu M. Varunan, Sunanda Bhattacharyya, Mrinal Kanti Bhattacharyya

**Affiliations:** 1 Department of Biochemistry, School of Life Sciences, University of Hyderabad, Hyderabad, Andhra Pradesh, India; 2 Department of Biotechnology, School of Life Sciences, University of Hyderabad, Hyderabad, Andhra Pradesh, India; Institut national de la santé et de la recherche médicale - Institut Cochin, France

## Abstract

Repairing double strand breaks (DSBs) is absolutely essential for the survival of obligate intracellular parasite *Toxoplasma gondii*. Thus, DSB repair mechanisms could be excellent targets for chemotherapeutic interventions. Recent genetic and bioinformatics analyses confirm the presence of both homologous recombination (HR) as well as non homologous end joining (NHEJ) proteins in this lower eukaryote. In order to get mechanistic insights into the HR mediated DSB repair pathway in this parasite, we have characterized the key protein involved in homologous recombination, namely TgRad51, at the biochemical and genetic levels. We have purified recombinant TgRad51 protein to 99% homogeneity and have characterized it biochemically. The ATP hydrolysis activity of TgRad51 shows a higher *K_M_* and much lower *k_cat_* compared to bacterial RecA or Rad51 from other related protozoan parasites. Taking yeast as a surrogate model system we have shown that TgRad51 is less efficient in gene conversion mechanism. Further, we have found that TgRad51 mediated gene integration is more prone towards random genetic loci rather than targeted locus. We hypothesize that compromised ATPase activity of TgRad51 is responsible for inefficient gene targeting and poor gene conversion efficiency in this protozoan parasite. With increase in homologous flanking regions almost three fold increments in targeted gene integration is observed, which is similar to the trend found with ScRad51. Our findings not only help us in understanding the reason behind inefficient gene targeting in *T. gondii* but also could be exploited to facilitate high throughput knockout as well as epitope tagging of *Toxoplasma* genes.

## Introduction

A broken chromosome if unrepaired leads to loss of genetic information and cell death. It is thus very important for a cell to identify and repair the damaged region. There are two major pathways by which double stranded DNA breaks (DSB) can be repaired. In homologous recombination (HR) mediated repair it relies on searching of extensive homologous stretches of DNA, whereas non homologous end joining (NHEJ) requires little or no homology. These two pathways compete with each other for repairing a DSB. It is a complex phenomenon and how cell decides which pathway to choose is not clear yet. Also different organisms show distinct usage frequency of HR over NHEJ during DSB repair. If a DSB is occupied by Ku70/80 hetero dimer, NHEJ follows. However, as soon as a DSB is formed, if the ends are resected by 5′-3′ exonuclease to form a long 3′ ss DNA, HR sets into action. This is because the long ssDNA overhang inhibits the loading of Ku70/80 and recruits the recombination proteins [1]. Depending upon the nature and orientation of the homologous sequences with respect to the break, a DSB can be repaired by any of the three different HR pathways: the single strand annealing (SSA), break induced replication (BIR) and the gene conversion (GC), also known as synthesis dependent strand annealing (SDSA) [2], [3], [4]. Presence of homology at both the DSB ends leads to GC. When homology is present only at one DSB end, BIR follows and when a DSB is flanked by direct repeats, SSA is the mechanism of choice. In the case of SSA, a minimum of 30 base pair homologous sequence on either side of the DSB is sufficient and such repair leads to the deletion of intervening sequences. All of the above mentioned pathways are driven by several proteins that belong to Rad52 epistatis group [5]. Strand invasion is the central step of HR mechanism and it requires Rad51 protein. However, Rad51 is essential only for GC, not for BIR or SSA [6], [7]. Rad51 protein has ATP dependent DNA binding activity, it multimerizes on single stranded DNA to form helical filament similar to that formed by bacterial RecA protein [8]. In an ATP hydrolysis dependent manner its motor activity searches for the homologous sequences between a single strand DNA and double stranded DNA and catalyses the strand exchange reaction. During this process it interacts with replication protein A (RPA), Rad52, Rad54 and Rad55 [9], [10], [11].

*Toxoplasma gondii* belongs to the eukaryotic phylum Apicomplexa, which infects about one third world population and under immune compromised condition it can cause serious illness. This protozoan parasite shares a number of structural similarities with disease causing parasites namely *Plasmodium*, *Cryptosporidium* etc. Thus it has become a model system to do functional genomics. However efficient gene targeting is a challenge in this parasite. It has been observed that in this parasite gene targeting efficiency is very low as they demonstrate high degree of non homologous end joining [12]. It is interesting to note that *T. gondii* is the only protozoan parasite that harbors non homologous end joining mediated DNA break repair mechanism. A divergent eukaryotic parasite *Trypanosoma brucei* possesses Ku70/80, however TbKu70/80 function in telomere maintenance [13] and Ku dependent NHEJ was not observed in *T. brucei* cell extract [14], [15]. The Ku70/80 heterodimer binds at the DSB along with DNA ligase IV-Xrcc4 complex and DNA-PKc to catalyze the break repair [16]. In a ku80 knock out background there is a 300–400 fold increase in targeted gene disruption in *T. gondii* [17]. The key protein, TgRad51, involved in targeted gene disruption has not been characterized yet. We have cloned, purified and characterized TgRad51 biochemically as well as genetically. It is observed that *T. gondii* and *Plasmodium falciparum* show strikingly opposite choice in DNA repair pathways. While *Plasmodium falciparum* depends solely on HR and apparently lacks NHEJ, *T. gondii* prefers NHEJ. Here we report the mechanistic insights for differential repair choices between these two closely related lower eukaryotes. PfRad51 has been identified as a DNA repair protein and has been speculated to play major role during mitotic recombination [18], [19]. PfRad51 has also been characterized biochemically [20] and despite having 82% identity in the catalytic domain, the kinetics of ssDNA dependent ATP hydrolysis activity differs markedly between PfRad51 and TgRad51. We hypothesize that compromised ATPase activity of TgRAD51 leads to inefficient gene targeting and poor gene conversion efficiency in *T. gondii*. However, with increase in homologous flanking ends, we observe an increase in targeted gene integration similar to the trend observed with ScRad51.

## Materials and Methods

### Yeast Strains

The yeast strains used in this work are tabulated in Table 1. The yeast expression vectors harboring *TgRAD51* and *ScRAD51* were transformed into the *Δrad51* (LS402) strain to create MVS26 and NRY2 strains respectively. The empty vector pTA [21] was transformed in *Δrad51* strain to generate NRY1. NA14 and NA14Δrad51 strains were used [4] for gene conversion study. We transformed the above mentioned yeast expression vectors with *TgRAD51* or *ScRAD51* to NA14Δrad51 strain to generate SSY1 and SSY2 strains respectively. To knockout Ku80 gene from a series of isogenic strains *KANMX* gene flanked by up-and down-sequences of *KU80* ORF was amplified from an already existing yeast *ku80Δ* strain. The primer pair OSB127 (AGT CTA TTA GCG GAA GTA CC) and OSB128 (GAA CGT CCT CTA CCC ACG) resulted in 200 bp and 220 bp flanking sequence of *KU80* on each side of *KANMX* gene. This linear *KANMX* cassette of 2220 bp was used to knockout *KU80* from the *rad51Δ* (LS402) strain. The yeast expression vector pTA was transformed into the *Δrad51Δku80* (SSY3) to generate SSY4 strain. The yeast expression vectors harboring *ScRAD51* and *TgRAD51* were transformed into the *Δrad51Δku80* (SSY3) strain to create SSY5 and SSY6 strains respectively.

**Table:** **Table1.** Yeast strains used in this study.

Strains	Genotype
W303a	*MATa 15ade2-1, ura3-1, 112 his 3-11, trp1, leu2-3*
NRY1	*MATa leu2-3,112 trp1-1 can1-100 ura3-1 ade2-1 his3-11,15 [phi+] RAD51:: LEU2 pTA*
NRY2	*MATa leu2-3,112 trp1-1 can1-100 ura3-1 ade2-1 his3-11,15 [phi+] RAD51::LEU2 pTA/ScRAD51*
MVS26	*MATa leu2-3,112 trp1-1 can1-100 ura3-1 ade2-1 his3-11,15 [phi+] RAD51::LEU2 pTA/TgRAD51*
NA14	*MATa-inc ura3-HOcs lys2::ura3-HOcs-inc* *ade3::GALHO ade2-1 leu2-3,112 his3-11,15 trp1-1 can1-100*
NA14Δrad51	*MATa-inc ura3-HOcs lys2::ura3-HOcs-inc* *ade3::GALHO ade2-1 leu2-3,112 his3-11,15 trp1-1 can1-100 RAD51::LEU2*
SSY1	*MATa-inc ura3-HOcs lys2::ura3-HOcs-inc* *ade3::GALHO ade2-1 leu2-3,112 his3-11,15 trp1-1 can1-100 RAD51::LEU2 pTA/ScRAD51*
SSY2	*MATa-inc ura3-HOcs lys2::ura3-HOcs-inc* *ade3::GALHO ade2-1 leu2-3,112 his3-11,15 trp1-1 can1-100 RAD51::LEU2 pTA/TgRAD51*
SSY3	*MATa leu2-3,112 trp1-1 can1-100 ura3-1 ade2-1 his3-11,15 [phi+] RAD51::LEU2 KU80::KANMX*
SSY4	*MATa leu2-3,112 trp1-1 can1-100 ura3-1 ade2-1 his3-11,15 [phi+] RAD51::LEU2 KU80::KANMX pTA*
SSY5	*MATa leu2-3,112 trp1-1 can1-100 ura3-1 ade2-1 his3-11,15 [phi+] RAD51::LEU2 KU80::KANMX pTA/ScRAD51*
SSY6	*MATa leu2-3,112 trp1-1 can1-100 ura3-1 ade2-1 his3-11,15 [phi+] RAD51::LEU2 KU80::KANMX pTA/TgRAD51*
SLY3	*MATa SBA1:: KANr*
Yku80Δ	*MATα leu2-3 112 trp1-1 can1-100, ura3-1 ade2-1 his 3-11,15 adh4::Ade2 rad5+ KU80::KANMX*
NKY8	*MATa SPC29-CFP:: KAN mRFP-TETR URA3::TETO GFP-LACI::LEU2 CHL1::HIS3*

### Construction of the pET: TgRad51 Expression Vector

The open reading frame encoding *Toxoplasma gondii* Rad51 was amplified from the cDNA library (provided by Prof. Vern Carruther) of *T. gondii* RH strain using hot start Kapa Hifi DNA polymerase (Kapa Biosystems) as described by the manufacturer. The forward primer OMKB65 (GGATCC ATGAGCGCCGTCTCTCTTCAG) and reverse primer OMKB82 (GGATCC TCAGTTGTCTTCGTAGTCGCC) are complementary to the 5′ and 3′ ends of the coding sequence of TgRad51 gene and the underlined oligo nucleotide represents the *BamHI* sites incorporated in both the primers. The PCR product of size 1065 bp was first cloned into TOPO2.1 TA vector (Invitrogen). The resultant plasmid was digested with *BamHI* to release the insert containing TgRad51 gene and was ligated with pET28a (Novagen) to construct N-terminal His_6_ tagged recombinant protein. The insert was sequenced and submitted to the Gene Bank (JQ771675). All primers used in this study were purchased from Sigma Aldrich.

### Plasmids

The *TgRAD51* gene was subcloned into *BamHI* site of the 2 µ expression plasmid, pTA under the control of GPD promoter. In a similar way, *ScRAD51* was PCR amplified from the genomic DNA isolated from W303a strain using the forward primer OMKB90 (GGATCC TGTCTCAAGTTCAAGAAC) and the reverse primer OMKB88 (CTGCAG CTACTCGTCTTCTTCTC), the underlined oligo nucleotides represented *BamHI* and *PstI* sites incorporated in the forward and reverse primer respectively. The PCR product of size 1203 bp was cloned into the yeast expression vector pTA under GPD promoter. All the cloning was confirmed by sequencing.

### Protein Purification

The expression vector pET28a:TgRad51 having N-terminal His_6_ tag was transformed into *Escherichia coli* host strain Rosetta (DE3). The whole transformation mixture was added to 10 ml LB media containing chloramphenicol and kanamycin and incubated at 37°C for overnight. Next morning 5% of the primary culture was added to 100 ml fresh LB media (containing chloramphenicol and kanamycin) and tested for the IPTG induced expression of the TgRAD51 protein. After OD_600_ reached 0.8, protein was induced by IPTG (Sigma) and grown on Luria broth for 4 hours at 37°C. About 1.0 gm cell pellet was suspended in 3 ml lysis buffer (50 mM NaH_2_PO_4_, 300 mM NaCl, 10 mM imidazole, 30% Glycerol, 20 mM β-mercaptoethanol) containing 1 mg/ml lysozyme and protease inhibitor (PMSF). It was then lysed on ice by sonication using (SONICS Vibra^m^ Cell™) giving ten 40 second bursts at 200 W with intermittent cooling. After lysis the cell debris was spun down by centrifugation for 45 minutes at 10,000 g at 4°C. The supernatant was applied to 0.75 ml 50% Ni-NTA agarose (Qiagen) and the loading flow through was collected. The resin was next washed with 16 volume of wash buffer (50 mM NaH_2_PO_4_, 300 mM NaCl, 20 mM Imidazole, 30% glycerol, 20 mM β-mercapitoethanol, 2% Tween 20). Further the column was washed with 5 volume of wash buffer 2 (50 mM NaH_2_PO_4_, 300 mM NaCl, 50 mM imidazole, 30% glycerol, 20 mM β-mercapitoethanol, 2% Tween 20). Finally the required protein was eluted first with buffer containing 250 mM imidazole (5 column volume) and then with 400 mM imidazole (50 mM NaH_2_PO_4_, 300 mM NaCl, 400 mM Imidazole, 30% glycerol, 20 mM β-mercapitoethanol, 2% Tween 20). The aliquots from the indicated steps in the elution profile were separated using 10% SDS-PAGE and visualized by Coomassie brilliant blue G-250 (Bio-Rad). The protein fractions eluted with 400 mM imidazole were pooled and dialysed against the dialysis buffer containing 20 mM Tris HCl (pH = 8), 1 mM dithiothretol (DTT), 5% glycerol. The concentration of the purified recombinant TgRad51 protein was determined by UV absorbance at 280 nm by using the extinction coefficient of 21025 M^−1^ cm^−1^ as calculated from the amino acid sequence by using ExPaSy Protparam tool. The purified protein was further confirmed by MALDI-TOF analysis and MS sequencing.

### ATPase Assay

The rate of ATP hydrolysis of TgRad51 was determined using Enz Chek Phosphate assay kit (Molecular Probe, E-6646). A typical reaction mixture was composed of 2 µM TgRad51 along with 30 fold molar excess of φxssDNA (New England Biolab) (60 µM) in presence of ATP containing reaction buffer (25 mM Tris-HCl pH 8, 5% glycerol, 1 mM DTT and 1 mM MgCl_2_). The reaction mixture was incubated at 37°C for 35 minutes and at every 5 minutes time interval a measured reaction volume was added (at 22°C) to purine nucleoside phosphorylase (PNP) reaction mix (manufacturer description) for 30 minutes. The ATP hydrolysis results in the formation of inorganic phosphate which was then reacted with MESG (2-amino-6-mercapto 7-methyl purine riboside) generating 2-amino-6-mercapto 7-methyl purine having an absorbance at 360 nm. This reaction is catalyzed by PNP. At a typical ATP concentration, the amount of inorganic phosphate released was plotted against different time interval and from the slope of the straight line, rate of the reaction was calculated. For determining the kinetic parameters the reaction was done at various concentrations of ATP (20 µM - 600 µM). The rate of the reaction was plotted using Graph pad Prism software against each concentration of ATP to determine the value of *K_M_* and *k_cat_*.

### Western Blotting

The expression of histidine tagged TgRad51 protein in bacterial cell was detected by using anti-His antibody (Santa Cruz Biotechnology Inc., CA). In order to detect endogenous level of TgRad51 and ScRad51 from NRY2, MVS26, SSY1 and SSY2, the protein was isolated according to the protocol as described in [21]. TgRad51 level was detected using anti PfRad51 antibody [18] in 1∶4000 dilution. The anti ScRad51 antibody (Santa Cruz Biotechnology Inc., CA) and anti Actin antibody (Abcam) were used in 1∶5000 dilution. Horseradish peroxide-conjugated rabbit IgG (Promega) was used as the secondary antibody for ScRad51 at 1∶10,000 dilution and HRP conjugated mouse IgG (Promega) was used as a secondary antibody for PfRad51 and actin at the same dilution. Proteins were visualized by an enhanced chemiluminescence system developed by the manufacturer (pierce). All the bands were normalized against Actin.

### Gene Conversion Assay

In NA14 strain, there is a cassette integrated in chromosome V where two consecutive *URA3* genes are separated by *KANMX6*. The first *ura3* gene is having a HO endonuclease recognition site in it. This strain expresses HO endonuclease when grown on galactose medium. Initially single colonies from each of the three strains NA14Δrad51, SSY1 and SSY2 were grown to logarithmic phase in the medium containing glycerol as a carbon source. About 1000 cells of each strain were then plated on two kinds of medium; one of them containing glucose and the other containing galactose as carbon sources. Colonies were counted on both the conditions after incubation for 3 days at 30°C. Galactose induced expression of HO creates a double stranded break at one of the *URA* locus and the repair efficiency was calculated by measuring the ratio of the number of colonies grown on the medium containing galactose, compared to that grown on medium containing glucose (no HO induction). Since such DSB repair could be achieved either by GC or SSA, in order to determine the efficiency of repair choice, we had replica plated the cells grown on galactose medium on the plates containing G418 sulphate. This assay was done thrice and the mean value was plotted using Graph Pad Prism5.

### Gene Targeting Assay

A 1536 base pair fragment containing *KANMX6* gene was excised from pFA6a-KANMX6 plasmid [22] using *NotI* restriction enzyme. This fragment was cloned at the *NotI* site of pSD158 plasmid [23], at the upstream of 5′ADH4 sequence. In this plasmid ADH4 gene was insertionally inactivated by introduction of *ADE2* gene. The 5′ADH4 and 3′ADH4 encompass 477 and 1527 base pair respectively. The cassette comprised of KANMX6-ADH4-ADE2-ADH4 was released from the modified pSD158 plasmid by *SalI* digestion which was subsequently used for gene targeting assay. This cassette was transformed to *Δrad51* (NRY1), NRY2 and MVS26, where efficient gene targeting at the ADH4 locus by homologous recombination resulted in expression of *ADE2* gene but did not confer resistivity to G418 sulphate. The transformed colonies were subsequently selected on SC-ade-trp plates. The colonies grown in individual strains were replica plated on G418 sulphate containing SC-trp plate. Colonies sensitive to G418 sulphate represents targeted integration of *ADE2* gene, while G418 sulphate resistant colonies correspond to random integration. Finally we calculated the % gene targeting efficiency by using the following formulae:


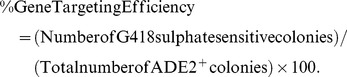


In each case the value obtained for *Δrad51* strain was subtracted. Each experiment was repeated three times. In another gene targeting assay, we monitored the gene targeting efficiency with increasing stretches of homologous flanking sequences (200 bp, 500 bp or 1000 bp). For that purpose we started with SLY3 strain where the *SBA1* gene was already insertionally inactivated by a *KANMX6* cassette [21]. We had amplified that *KANMX6* cassette by three different sets of primers positioned at 200 bp/500 bp/1000 bp upstream and 200 bp/500 bp/1000 bp down stream of the *KANMX6* insertion. The primer pair OSB5 (TGCTACCCGCCTTCGAGTG) and OSB6 (CACATACAGTTCCATTACTTGAC) resulted in 200 bp flanking sequence on each side of *KANMX6* cassette. Similarly OMKB193 (CTCAGAAGAATTTCGTAAATCGG) and OMKB194 (GGAGATGGTACCGGTTAAGCG) produced 500 bp flanking sequences of *SBA1* on either side of *KANMX* and OMKB191 (TCACACGTCCGTCATGTCTAC) and OMKB192 (GTCCTGCAGGAGACTTATTAGC) amplified 1 Kb flanking regions on either side of *KANMX6* cassette. These three different PCR products were individually transformed to *Δrad51* (NRY1), NRY2 and MVS26 strain to knock out *sba1* by homologous recombination and selected on SC-trp containing G418 sulphate. The number of G418 sulphate resistant colonies was measured. We compared the efficiency of targeted integration in the *rad51Δ* and *rad51Δku80Δ* strains. The gene targeting efficiency was monitored in these strains background with increasing length of homologous flanking sequences (500 bp or 1000 bp). In NKY8 strain, the *CHL1* gene was already insertionally inactivated by a *HIS* cassette. We had amplified the *HIS* cassette by two sets of primers positioned at 500 bp/1000 bp upstream and 500 bp/1000 bp downstream of the *HIS* insertion. The primer pair OMKB 211 (CAG CTC TCT AGC CAA CAG CAG) and OMKB 212 (CTT GCG TAT TAT CTA TAG CGG C) resulted in 500 bp flanking sequence on each side of *HIS3* marker. Similarly OMKB 213 (CAC TCG TTG ACT AGT TCA GAG G) and OMKB 214 (GAC GAA CTT CAT GTG ACG GCT G) produced 1 Kb flanking sequences on each side of the *HIS3* marker. These two PCR products were individually transformed into NRY1, NRY2 and MVS26 strain to knock out *CHL1* gene by homologous recombination and the transformants were selected on SC-trp-his plate. The numbers of colonies were measured. Similar experiments were done with SSY4, SSY5 and SSY6 strain to replace *CHL1* gene with *HIS3* marker by homologous recombination and the transformants were selected on Sc-Trp-His plates. The numbers of His+ colonies were counted. Integration at the targeted loci was determined by PCR. Each experiments were repeated at least three times and was plotted using Graph Pad Prism with error bars.

## Results

### Identification of *Toxoplasma gondii* Rad51 Open Reading Frame

**Figure 1:**
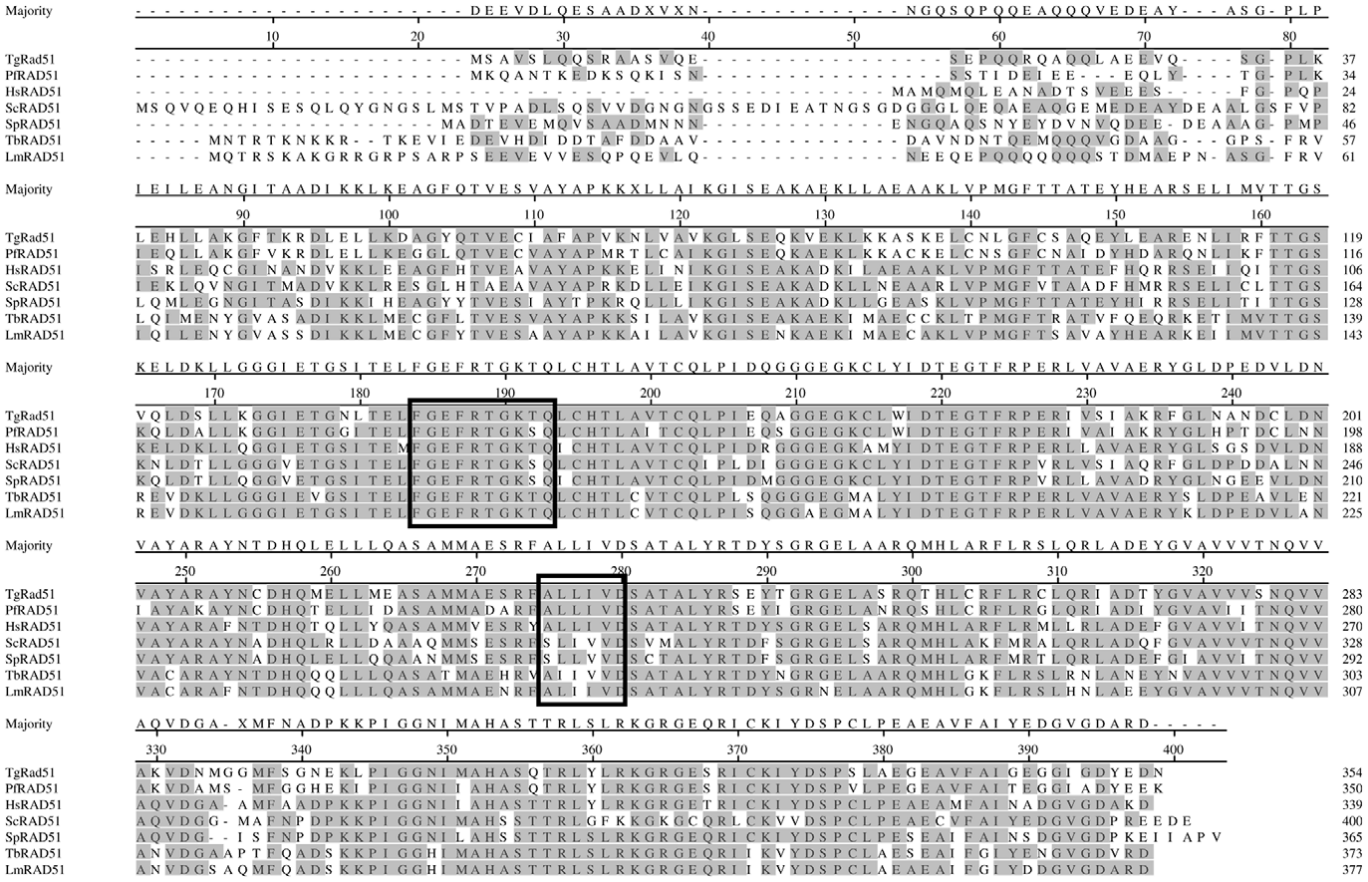
Alignment of TgRad51 with *Plasmodium falciparum* (PfRad51), human (HsRad51), *Saccharomyces cerevisiae* (ScRad51), *Schizosaccharomyces pombe* (SpRad51), *Trypanosoma bricei* (TbRad51) and *Leishmania major* (LmRad51) using Clustal method (Meg align, DNA star). Shaded areas represent identical amino acids. The two DNA binding Walker motifs are boxed.

A BLAST search of ToxoDB database (www.toxodb.org) have identified three ORFs corresponding to putative RAD51 orthologs from ME49 strain (TGME49_072900), VEG strain (TGVEG_020310) and GT1 (TGGT1_112080) strain. Based on these sequences we have designed PCR primers and amplified TgRad51 ORF from RH strain using cDNA library as template (a kind gift from Dr. Vern Carruthers). Sequence comparison of the amplified ORF indicated that it is an ortholog of Rad51. We found little sequence polymorphism of RAD51 gene among the four strains of *T. gondii* (Figure S1). The deduced amino acid sequence of TgRad51 protein contains 354 amino acids with a predicted molecular mass of 38,796 and isoelectric point (pI) value of 5.77. Further sequence comparison of TgRad51 protein with other known eukaryotic Rad51 orthologs revealed a high degree of overall identity (52 to 72%) and even higher degree of conservation (65 to 82%) in the core catalytic domain. Computer alignment using Clustal method (Meg align, DNA star) revealed a near perfect alignment and identified the two nucleotide binding Walker motifs (Figure 1). Phylogenetic analysis revealed that TgRad51 is closer to another apicomplexan parasite PfRad51.

### Purification of Recombinant TgRad51 Protein

**Figure 2:**
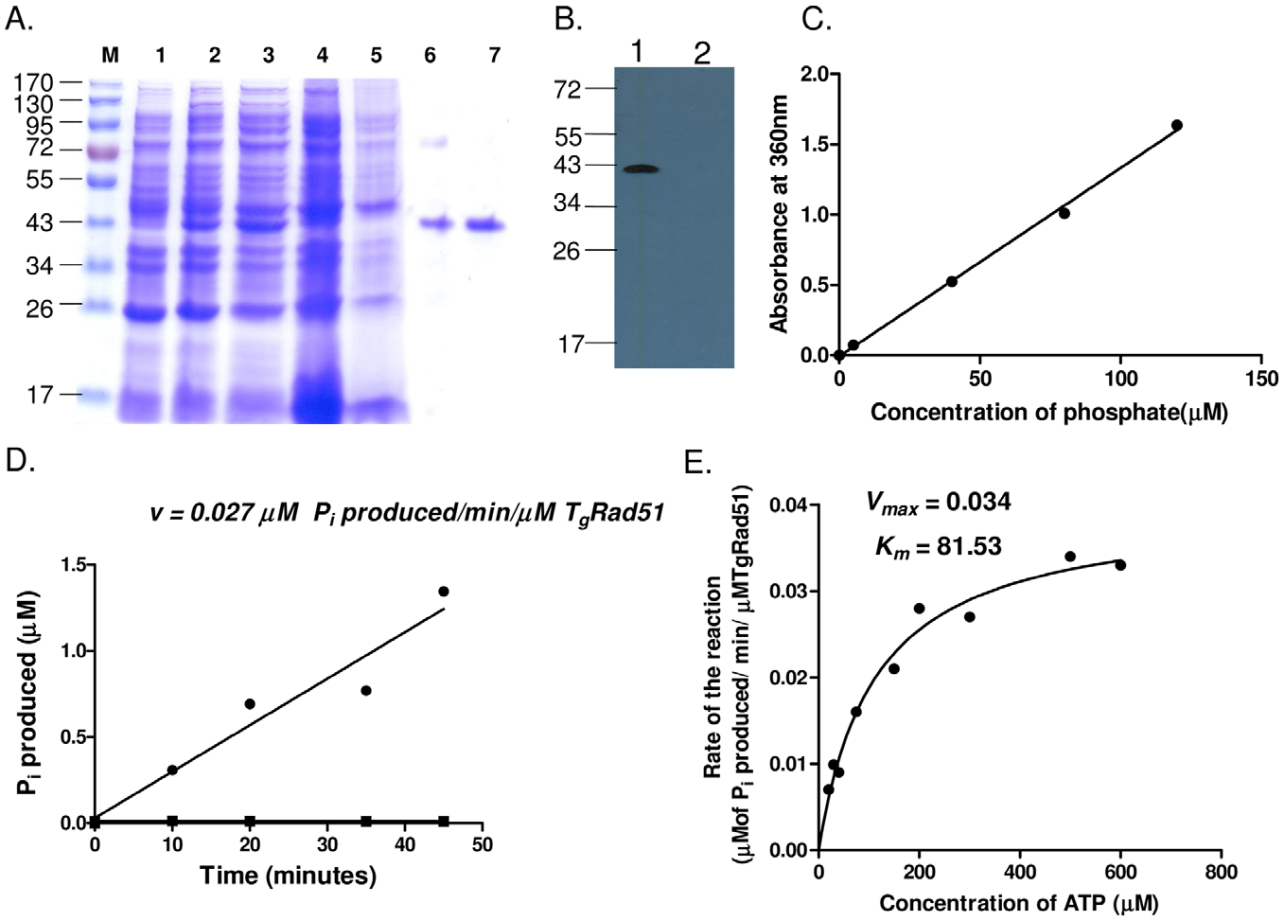
Purification and ssDNA dependent ATPase activity of recombinant TgRAD51 protein. (A) M corresponds to molecular weight standards, the sizes of marker proteins in kDa are indicated, Lane 1 and 2 are uninduced and induced cell free extracts respectively, Lane 3 corresponds to the proteins in soluble fraction, Lane 4 being the loading flow through and Lane 5 is the washing flow through. Lane 6 and 7 correspond to the eluted fractions with increasing imidazole concentrations i.e. 250 mM and 400 mM respectively. B) Western blot analysis shows the presence of Histidine tagged TgRAD51 in lane 1 from the cells bearing pET-TgRAD51 and lane 2 shows the absence of the protein in the cell bearing the empty vector pET. C) The standard curve showing linear relationship between inorganic phosphate added and increase in absorbance at 360 nm in response to the addition to MESG as substrate and the enzyme PNP (EnzChek phosphate assay kit). D) ssDNA dependent ATP hydrolysis of TgRAD51 using EnZChek phosphate assay kit. At 300 µM ATP concentration, with 2 µM TgRAD51 and 60 µM φxssDNA, the rate of the reaction as calculated from the slope of the curve is 0.027 µM P_i_ produced/min (•) where as in absence of ssDNA (▪) no ATP hydrolysis occurs. E) Michaelis Menten curve has been plotted with ATP concentrations in the range of 20 µM to 600 µM and kinetic parameters are derived from the curve.

The expression system Rosetta (DE3)/pET:TgRad51 was used for the purification of TgRad51 protein. The expression vector pET:TgRad51 was transformed into Rosetta (DE3) cells, which has the pRARE plasmid that supplies the tRNA for six codons that are rarely used in *E. coli* (AUA, AGG, AGA, CUA, CCC and GGA). Figure 2A (lane 2) shows that TgRAD51 protein corresponds to about 25% of the total proteins in the crude cell extract after IPTG induction. The induced recombinant protein was predominantly detected in the soluble fraction (lane 3). Figure 2A shows the purification profile of recombinant TgRad51 protein through Ni-NTA column. Glycerol (30%) was included in the wash buffer in order to remove the unwanted hydrophobic interactions between non specific proteins attached with TgRad51. The elution at 250 mM imidazole results in about 90% purified protein with minor protein contaminants (lane 6). However, 400 mM imidazole containing elution buffer resulted in greater than 99% purified protein as judged by SDS-polyacrylamide gel electrophoresis (lane 7). Finally the purified protein was dialyzed thoroughly against Tris-HCl (pH 8) to remove excess imidazole.

Mass spectroscopic analysis confirmed the presence of TgRad51 protein and yielded a molecular mass of 39,398 Da in agreement with the molecular mass of 38,796 Da predicted by ExPasy Protparam tool. MS-MS analysis yielded sequence of 5 peptides corresponding to the predicted amino acid sequence of TgRad51 (Figure S2). The western blot analysis with anti histidine antibody confirmed the presence of purified TgRad51 (Figure 2B).

### ssDNA Dependent ATP Hydrolysis of TgRAD51

In order to investigate whether the low gene targeting efficiency in *T. gondii* is due to a compromised Rad51 protein activity, we determined the ATP hydrolysis activity of purified TgRad51 protein. We used EnzChek Phosphate Assay kit (Molecular Probes) to determine the rate of ATP hydrolysis of TgRad51. In this assay the inorganic phosphate when combines with the substrate MESG (2-amino-6-mercapto-7-methylpurine riboside) in presence of the enzyme PNP (purine nucleoside phosphorylase) produces ribose 1-phosphate and 2-amino-6-mercapto-7-methylpurine. Formation of the product changes the absorbance of the substrate from 330 nm to 360 nm which can be measured spectroscopically. Figure 2C shows the steep increment of the absorbance at 360 nm with increasing concentration of the inorganic phosphate. When we incubated TgRad51 with ATP at 37°C in presence of φxssDNA, ATP is hydrolyzed to release phosphate. At different time interval aliquots of this reaction mixture were withdrawn and added to a second reaction mixture containing MESG along with PNP. Accordingly we monitored the kinetics of ATP hydrolysis of TgRAD51 in presence of ssDNA. Figure 2D shows that TgRAD51 is incapable of ATP hydrolysis in absence of ssDNA. However in presence of 30 fold molar excess of ssDNA, ATP hydrolysis took place. At 300 µM ATP concentration, the rate of ATP hydrolysis as calculated from the slope of the curve was found to be 0.027 µM/min. The ATPase activity of TgRad51 was measured at different ATP concentrations and used to plot Michaelis Menten curve (Figure 2E). The data was also used to generate Lineweaver Burk plot. The *k*_cat_ value (0.034 min^−1^) and *K*_m_ value for TgRad51 (81.53 µM) were calculated using Graph Pad Prism. Our results indicate that the ATP hydrolysis activity of TgRad51 protein is indeed very weak.

### Gene Conversion Efficiency of TgRAD51 is Poor Compared to that of ScRAD51

**Figure 3:**
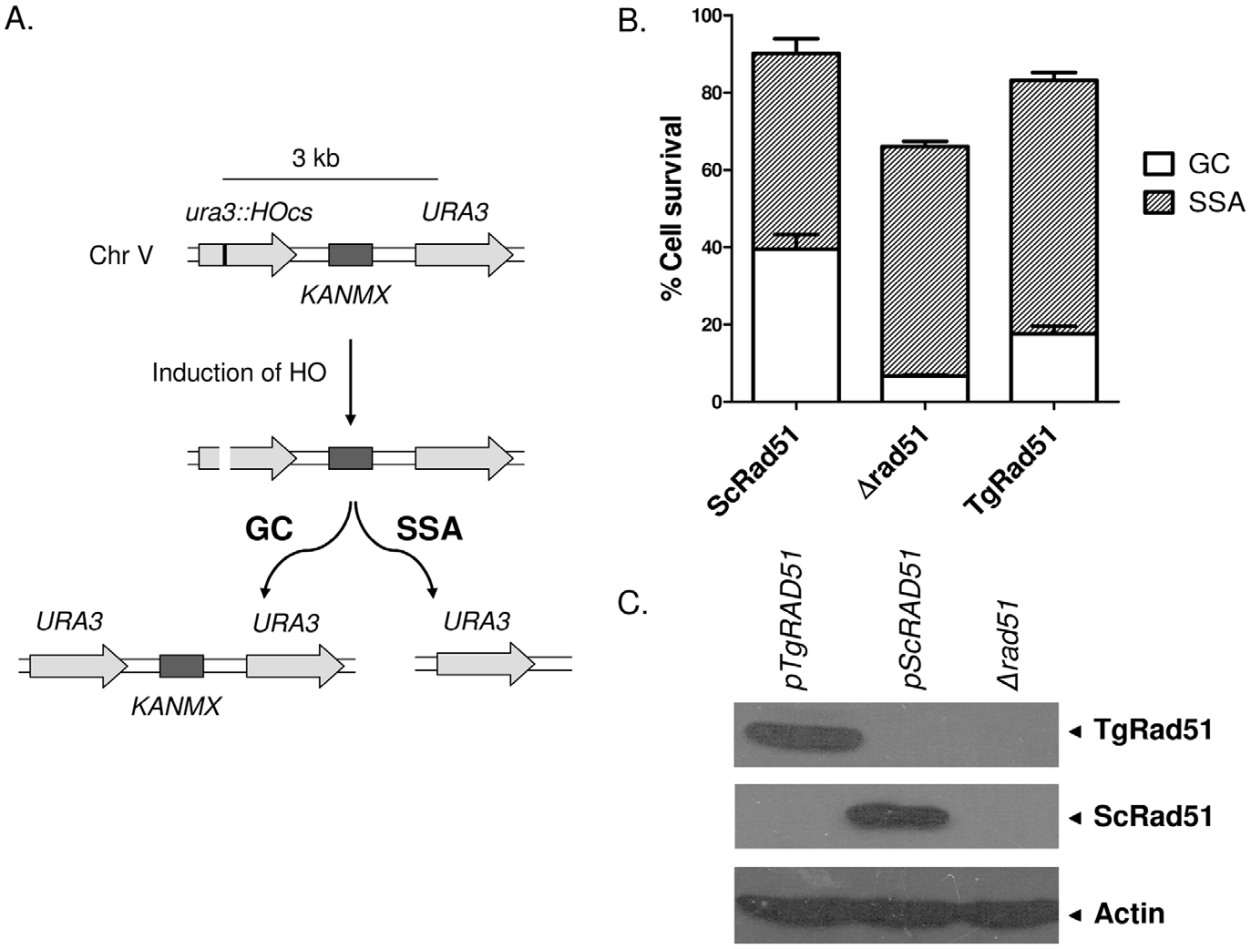
Gene conversion efficiency of *TgRAD51* is poor compared to that of *ScRAD51*. (A) Schematic diagram of DSB repair choice experiment. *URA3* and *KANMX* represent wild type alleles. A HO endonuclease site is incorporated in *ura3::HOcs* mutant allele. Once the HO induced DSB is repaired by gene conversion (GC), the mutant *ura3::HOcs* allele is converted into wild type *URA3* allele. A single strand annealing (SSA) event leads to deletion of the intervening sequence (containing *KANMX* gene) and merging of *ura3::HOcs* and *URA3* alleles to create the wild type *URA3* allele. (B) Bar diagram showing the percentage of cells survived after induction of DSB. The relevant genotypes are marked on the X-axis. The white bars represent fraction of the cells survived by repairing the DSB using GC mechanism, where as the hatched bars denote the fraction of the survivors that employed SSA mechanism. Each bars represent mean value ± SD from 4 different experiments. (C) Western blots showing the abundance of ScRad51 and TgRad51 proteins. Different lanes are marked with the respective genotypes. Actin is the loading control.

The ATP hydrolysis activity of Rad51 protein is essential for its motor activity, which is instrumental for the genome-wide search for a homologous template during the pre-synapsis stage of homologous recombination. In order to investigate how the weak ATP hydrolysis activity of TgRad51 affects homology search, we have assayed gene conversion (GC) efficiency of TgRad51. Repair of a DSB by GC relies on Rad51 mediated homology search. To this end we have used yeast as a surrogate model system where GC could be assayed accurately and quantitatively. We used a yeast strain NA14 having two copies of *URA3* gene in chromosome V with a *KANMX6* selectable marker in between (Figure 3A). One of the *ura3* genes is made nonfunctional by incorporating HO endonuclease site in it. The functional *URA3* gene is present in the same chromosome about 3 kb apart. The strain harbors *HO* endonuclease from GAL1 promoter, which is activated by growing the cells in presence of galactose as a carbon source. When a DSB is generated in *ura3* gene by induction of *HO* endonuclease it can be repaired either by GC using the homologous template (*URA3*) present in the same chromosome 3 kb apart, or it can be repaired by single strand annealing (SSA), which results in deletion of a stretch of intervening DNA sequence. We created two strains SSY1 and SSY2 by transforming the expression vector pTA*ScRAD51* and pTA*TgRAD51* respectively to *NA14*Δ*rad51* strain. In order to assay the efficiency of DNA repair activity of *TgRAD51* we created a single DSB in the *ura3* gene by HO induction and counted the number of cell survival on galactose containing plate and compared to the number of cells on glucose containing plate. Our result shows that the repair efficiency of TgRAD51 (83%) is comparable to that of the wild type (96%), while *NA14*Δ*rad51* being 63% (Figure 3B). In order to monitor the contribution of GC among the survivors, we looked for G418 sulphate resistant cells. If the DSB is repaired by GC, the survivor colonies will be G418 resistant. On the other hand if the break is repaired by SSA, the survivor colonies will be G418 sensitive due to the loss of *KANMX6* cassette. We found that the majority of the survivors (77%) of SSY2 strain (TgRad51) are due to repair by SSA, which does not require the presence of Rad51 protein, gene conversion efficiency being only 23% of the total. On the contrary SSY1 strain (ScRad51) exhibited almost equal preferences for GC and SSA pathways, i.e. approximately 42% of its survival by GC and 58% by SSA (Figure 3B). Hence the GC efficiency of SSY1 (ScRad51) strain is about two fold more than that of SSY2 (TgRad51) strain. The key protein involved in homologous recombination mediated gene conversion is the Rad51 and our result shows that TgRad51 possess inefficient gene conversion activity compared to ScRad51. To rule out the possibility that inefficient gene conversion is not due to poor expression of TgRAD51 in heterologous system, we have done Western blot analysis of SSY1 and SSY2 strains and probed with anti ScRAD51 and PfRAD51 antibodies respectively (anti-PfRad51 antibody cross-reacts with TgRad51 protein). Our data (Figure 3C) shows that TgRad51 expression is comparable to that of ScRad51, actin being the loading control. We conclude that low ATPase activity of TgRad51 is responsible for poor gene conversion efficiency.

### Gene Targeting Efficiency of TgRAD51 Increases with Increase in Stretch of Homologous Sequences

**Figure 4:**
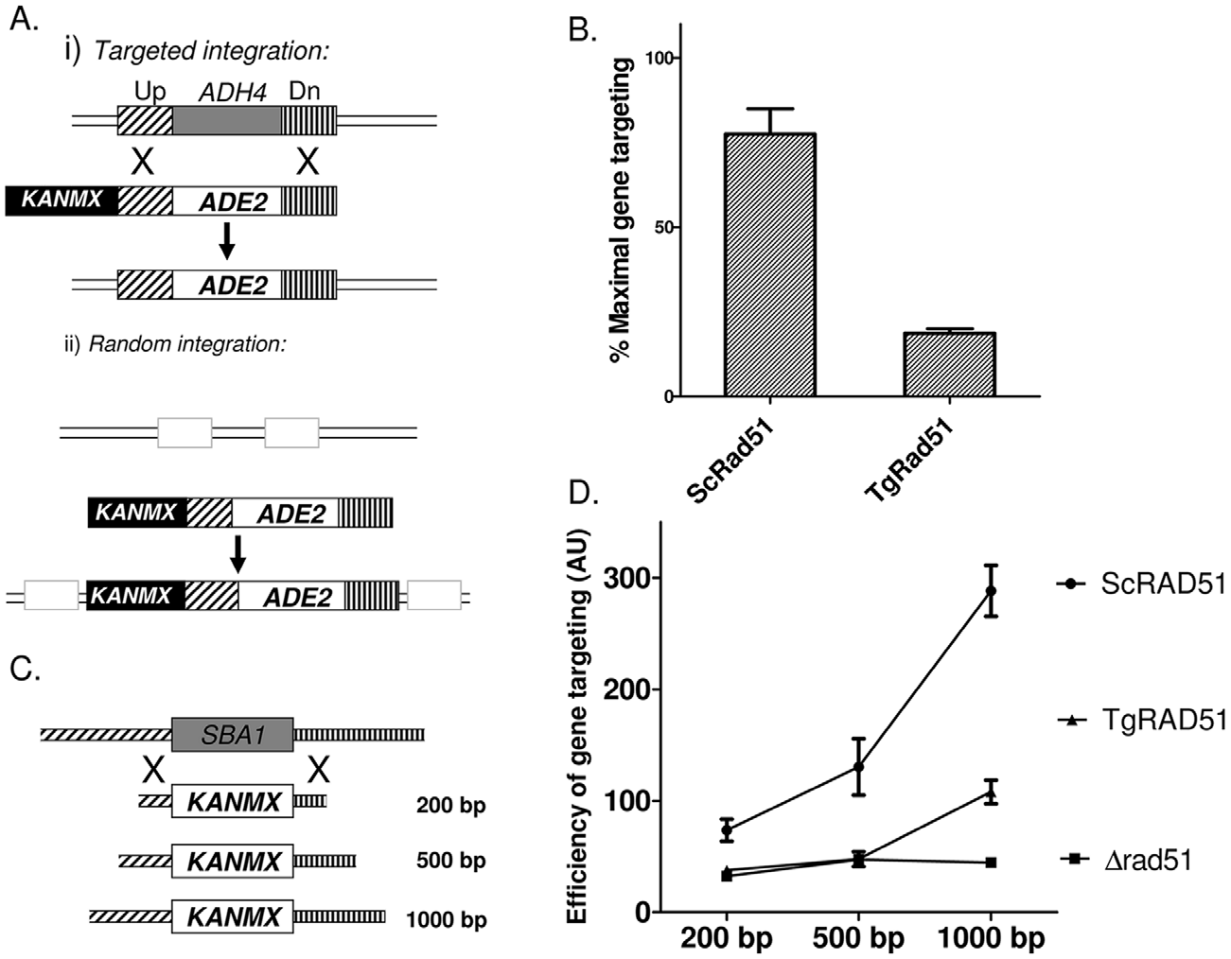
Gene targeting efficiency of TgRAD51 increases with increase in stretch of homologous sequences. (A) Schematic diagram showing molecular events leading to targeted integration of ADE2 gene at ADH4 locus versus random integrations. KANMX is a second selectable marker retained only in case of random integration via non-homologous recombination mechanism. (B) Bar diagram showing efficiency of gene targeting at the ADH4 locus in cells harboring ScRad51 or TgRad51 as the sole recombinase. The mean value from four independent experiments is plotted with standard deviations. (C) Schematic diagram showing knockout strategy for *SBA1* gene. Varying lengths of flanking homologous sequences on either side are indicated. (D) Efficiency of gene knockout with increasing flanking homology. The lengths of the flanking homologous stretches are indicated on the X-axis. These experiments are done at least 3 times and the mean values with standard deviations are plotted.

A linear piece of DNA bearing homology to a part of the genome can either be integrated at the correct chromosomal locus by means of homologous recombination, or it could be integrated at any random site on the chromosomes via non-homologous recombination. In *T. gondii* targeted integration is extremely less efficient. We wanted to investigate whether such less efficient gene targeting is due to the weak ATP hydrolysis activity of TgRad51, since gene targeting at the correct locus demands search of two homologous needles in the genomic hay stack. In order to appreciate the contribution of TgRad51 protein alone, from other *Toxoplasma* proteins presumably involved in HR pathway, we have used a surrogate yeast model, where the yeast *RAD51* gene is deleted and is replaced by *TgRAD51* gene. Thus, in this system, the main recombination protein (Rad51) is from *T. gondii* and all other auxiliary proteins are from yeast origin. We have engineered this system in such a way that it would lead us to investigate the frequency of targeted vs. random integration choices in the cell harboring TgRAD51. The integration construct contains two selectable markers: *ADE2* and *KANMX6*; while the *ADE2* marker scores for all the integration events (targeted or random), the *KANMX6* marker is able to differentiate between random versus targeted integration. In case of targeted integration at the *ADH4* locus the *KANMX6* marker will be lost as a result of homologous recombination. On the other hand, during NHEJ mediated random integration the *KANMX6* cassette will be retained and such transformants will be G418 resistant. Thus random integration of the cassette would result in ADE2^+^ and G418^R^ colonies (Figure 4Aii), whereas the targeted integration would lead to ADE2^+^ and G418^S^ colonies (Figure 4Ai).

We transformed the KANMX-ADH4-ADE2-ADH4 cassette in three strains: NRY1 (Δ*rad51* containing an empty vector pTA); NRY2 strain (Δ*rad51* containing an expression vector pTA: *ScRAD51*); and MVS26 strain (Δ*rad51* containing an expression vector pTA: *TgRAD51*). The experiments with three repeats showed that NRY2 cells (*ScRAD51*) prefer to go for targeted integration at the ADH4 locus with about 85% frequency, where as *TgRAD51* showed a preference for random integration having only 24% frequency for targeted integration (Figure 4B). We hypothesize that poor ATP hydrolysis activity of TgRad51 results in inefficient motor activity of the TgRad51 protein, as a result of which it shows three times reduction in gene targeting efficiency compared to that of *ScRAD51*. A prediction of this hypothesis would be that the increment of flanking homologous sequences would facilitate the homology search and thus would compensate for the weak ATP hydrolysis of Rad51. So, the next question we asked was whether the increase in homologous stretch of DNA has any effect on the frequency of TgRad51 protein mediated targeted integration. To test this hypothesis we designed three constructs to knockout *SBA1* gene. These constructs contains a selectable marker *KANMX6* flanked by upstream and downstream sequences (200 bp/500 bp/1000 bp on either side) of SBA1 gene (Figure 4C). These three constructs were used to disrupt the *SBA1* gene in the cells bearing *ScRAD51* (NRY2) and *TgRAD51* (MVS26). As expected, our result showed that with the increase in homologous sequence there was a gradual increase in the frequencies of the targeted gene disruption (Figure 4D). NRY1 (*Δrad51)* cells showed very less number of targeted disruptions as measured by the survival on G418 sulphate plates throughout varied degree of homology. With increase in flanking homologous sequences, NRY2 cells (*ScRAD51*) showed a steep rise in the gene targeting efficiency (Figure 4D). MVS26 (*TgRAD51*) cells behaved much like *Δrad51* strain up to 500 base pair flanking homology. As the homologous stretches were increased to 1000 base pairs on either side of *KANMX6*, the number of G418^R^ colonies also increased sharply for MVS26 strain. The increase in homologous flanking region causes same fold increase (approximately 2.5 fold) in the gene targeting efficiency for both *ScRAD51* and *TgRAD51*.

### Gene Targeting Efficiency of TgRad51 is Independent of Ku80 Function

**Figure 5:**
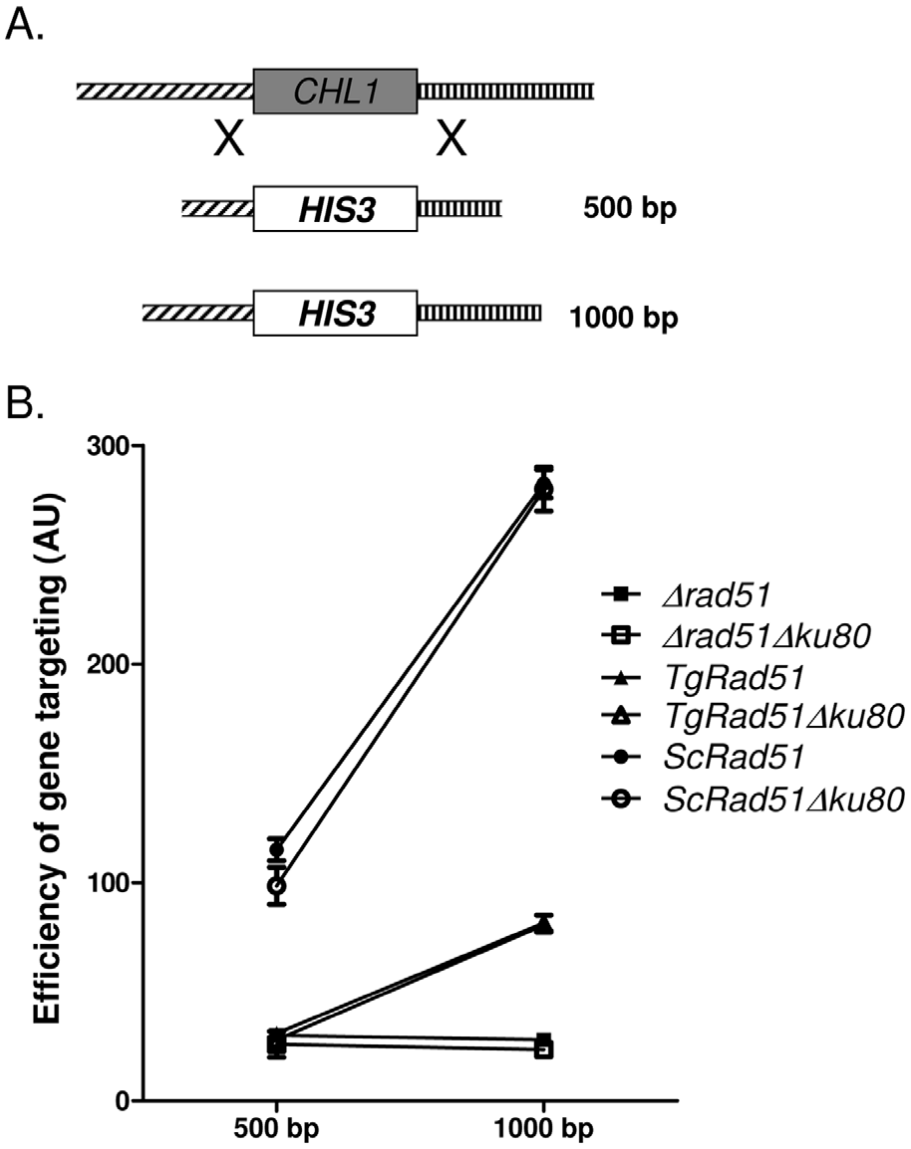
Gene targeting efficiency of TgRad51 is independent of Ku80 function. (A) Schematic diagram showing knockout strategy for *CHL1* gene. Varying lengths of flanking homologous sequences on either side are indicated. (B) Efficiency of gene knockout with increasing flanking homology in *KU80* proficient (closed- circle, triangle or square) and KU80 deficient (open- circle, triangle or square) cells. The lengths of the flanking homologous stretches are indicated on the X-axis. These experiments are done at least 3 times and the mean values with standard deviations are plotted.

In order to investigate whether efficiency of Rad51 mediated gene targeting is affected by the absence of Ku80 protein, we have compared gene targeting at the *CHL1* locus in presence or in absence of Ku80. To this end we have tested six strains: MVS26 (*TgRAD51*); SSY6 (*TgRAD51* Δ*ku80*); NRY2 (*ScRAD51*); SSY5 (*ScRAD51* Δ*ku80*); NRY1 (Δ*rad51*); and SSY4 (Δ*rad51* Δ*ku80*). To disrupt *CHL1* gene we have generated two constructs. These constructs contain a selectable marker (*HIS3*) flanked by upstream (500 bp/1000 bp) and downstream (500 bp/1000 bp) sequences of *CHL1* ORF (Figure 5A). In accordance to our findings with gene targeting at the *SBA1* locus, we observed that the larger the flanking homology the greater is the gene targeting efficiency at the *CHL1* locus. When compared between the *KU80* proficient and *KU80* deficient cells, we find no significant change in gene targeting efficiency. This observation holds true for both ScRad51 and TgRad51 mediated gene targeting (Figure 5B). Thus, we conclude that gene targeting efficiency is independent of Ku80 function.

## Discussion

Several important findings of this study collectively help us in understanding the DSB repair pathways in *T. gondii* as a whole and the underlying reasons for inefficient gene targeting in particular. We have cloned, expressed and purified recombinant TgRad51 and have shown that it possesses ATP hydrolysis activity, which is the lowest among all the eukaryotic Rad51 proteins studied so far. Using yeast as a surrogate system we have characterized *TgRAD51* genetically. To this end we have performed three independent experiments: repair choice experiment, targeted gene knock-in experiment and targeted knock-out experiment. In the repair choice experiment the induced DSB created within *URA3* gene is repaired by any of the two HR mechanisms: gene conversion (GC) or single strand annealing (SSA). While SSA does not depend on Rad51, GC totally depends on Rad51 mediated homology search. Our finding that only GC is compromised in cells harboring TgRad51 is thus consistent. Two other assays (namely gene knock-in and gene knock-out) that also depend on Rad51 mediated homology search are also found to be affected by TgRad51.

Double strand breaks (DSBs) can cause damage to the genomic integrity of a cell. Repair of such DSBs are essential for cell survival. A DSB can be repaired either by homologous recombination (HR) or by non-homologous recombination (NHEJ). Prokaryotes and lower eukaryotes prefer high-fidelity repair mechanism such as HR, where as higher eukaryotes show preference towards mutagenic NHEJ pathway. An attractive hypothesis links the repair choice to gene density. Due to the low gene density in higher mammals mutations acquired during NHEJ mediated repair of random DSBs, are less likely to affect gene function, where as in organisms with high gene density, the likelihood of having random DSBs within the genes are higher and concomitantly repair of such breaks by NHEJ might affect gene function. For example there are about 7 genes per mega base of mouse or human genome, thus the probability of having random breaks in the intergenic regions are higher than within the genes. On the other hand prokaryotes and lower eukaryotes have very high gene density. The gene densities (number of genes per mega base) of *E. coli*, *S. cerevisiae*, *S. pombe*, *Leishmania major* and *Plasmodium falciparum* are 952, 465, 396, 297 and 231 respectively. Thus it is not surprising that all the aforementioned organisms prefer HR over NHEJ. The recombinase RecA (Rad51 ortholog) from most of these organisms have robust ATP hydrolysis activity (the *k_cat_* values for EcRecA, ScRad51, and LmRad51 are 18 min^−1^, 2.9 min^−1^, 0.7 min^−1^) [24], [25], [26] compared to that of mammals (*k_cat_* of hRad51 is 0.16 min^−1^) [27]. Thus, a strong ATPase activity of Rad51 is an indicative of a robust HR system. This notion is supported by studies with mutant hRad51 (K133R) incapable of ATP hydrolysis, where gene targeting is severely compromised. However, this mutant does not have any effect on strand exchange between homologous templates. Moreover, the DNA repair ability of the mutant cell is not affected [28]. *T. gondii* despite being a lower eukaryote shows preference to NHEJ pathway. The low gene density of *Toxoplasma* genome (100 genes per mega base) and our finding that TgRad51 too have a weak ATP hydrolysis activity (*k_cat_* is 0.034 min^−1^) fits very well with the choice of DSB repair pathway in this organism. Although, it appears that HR might not be a general mechanism for repair of DSB in *T. gondii*, whether HR activity is required to repair specific types of DSB ends remains as an open question. It is likely that the primary function of HR in *T. gondii* could be during sexual reproduction.

TgRad51 is the first member of the recombination machinery of *T. gondii* to be characterized. The existence of putative orthologues of Dmc1, Rad54, Rad50 and Mre11 in *Toxoplasma* genome suggests that this parasite does possess a functional recombinosome. Interestingly, the apparent lack of Rad52 orthologue in *T. gondii, Plasmodium falciparum* and *Cryptosporidium parvum* is suggestive of a Rad52 independent recombination mechanism in these apicomplexan parasites.

Gene disruption and epitope tagging are the two most important tools to study gene function in the post genomics era. Both of these techniques rely on homologous recombination mechanism. Since the HR machinery is weak in *T. gondii*, targeted gene disruption or tagging of endogenous genes are very less efficient in this parasite. Works from Bzik laboratory and Carruthers laboratory have demonstrated enhanced gene targeting in *ku80* null *T. gondii* [17], [29]. However, as expected there is no change in gene targeting efficiency in *ku80* null parasite lines [17]. In our assay we used yeast as surrogate organism to investigate whether the absence of Ku80 protein has any positive effect on the efficiency of gene targeting. Since it is possible to obtain individual clonal transformants on solid medium using yeast system, it allowed us to determine the locus of integration (targeted versus random) in each of the individual colonies. We found that the presence or absence of Ku80 protein does not have any significant effect on gene targeting efficiency per se. This is because, the absence of Ku80 abrogates NHEJ pathway but does not increase the efficiency of Rad51 recombinase. An organism that harbors these two competing pathways (HR and NHEJ) may results in either random integration of the transfected DNA *via* NHEJ mechanism or integration at the right locus *via* HR mechanism. Only one of the two types of recombinant clones (where integration took place at the right locus) is desirable. Abrogating NHEJ pathway (by knocking out *KU80* or other molecular players of NHEJ) would definitely minimize the number of those recombinant clones where random integration has taken place and thereby facilitate the screening of the desired clone. However, it would not increase the integration efficiency at the correct locus. This notion is supported by the finding that targeted repair of Δ*hxgprt* became independent of TgKu80 when enough flanking homology (910 bp) was provided [17]. Genome wide homology search by ssDNA bound Rad51 is the rate determining step in homology directed recombination. Thus, longer homologous stretches are likely to have great positive impact on such process. Our finding that increased flanking homology facilitate targeted gene knock out by TgRad51 is in corroboration with this hypothesis. Similarly, since ATP hydrolysis activity of Rad51 is required for such homology search a higher ATPase activity also results in more efficient gene targeting. It will be interesting to see whether a transgenic *T. gondii* harboring Rad51 from *L. major* or *S. cerevisiae* could significantly increase the gene targeting efficiency in this protozoan parasite. Future studies might test this possibility either by expressing LmRad51/ScRad51 (having robust ATPase activity) in *T. gondii* from an episomal plasmid or by delivering purified Rad51 proteins *via* nano particle mediated delivery systems. Positive results from such studies will have a great impact on functional studies of Toxoplasma biology.

## Supporting Information

Figure S1 Phylogenetic analysis of eukaryotic Rad51 proteins using Clustal method (Meg align, DNA star). T. gondii Rad51 is highlighted.
(TIF)

Figure S2 Amino acid sequence of TgRad51 protein from RH strain. The underlined peptide sequences were generated from MS-MS analysis.
(TIF)
