# Supplementary figures and images for: Characterization of Rad51 from Apicomplexan Parasite Toxoplasma gondii: An Implication for Inefficient Gene Targeting
Source: PLoS One. 2012 Jul 30;7(7):e41925. doi: 10.1371/journal.pone.0041925 (PMC3408395; doi:10.1371/journal.pone.0041925)

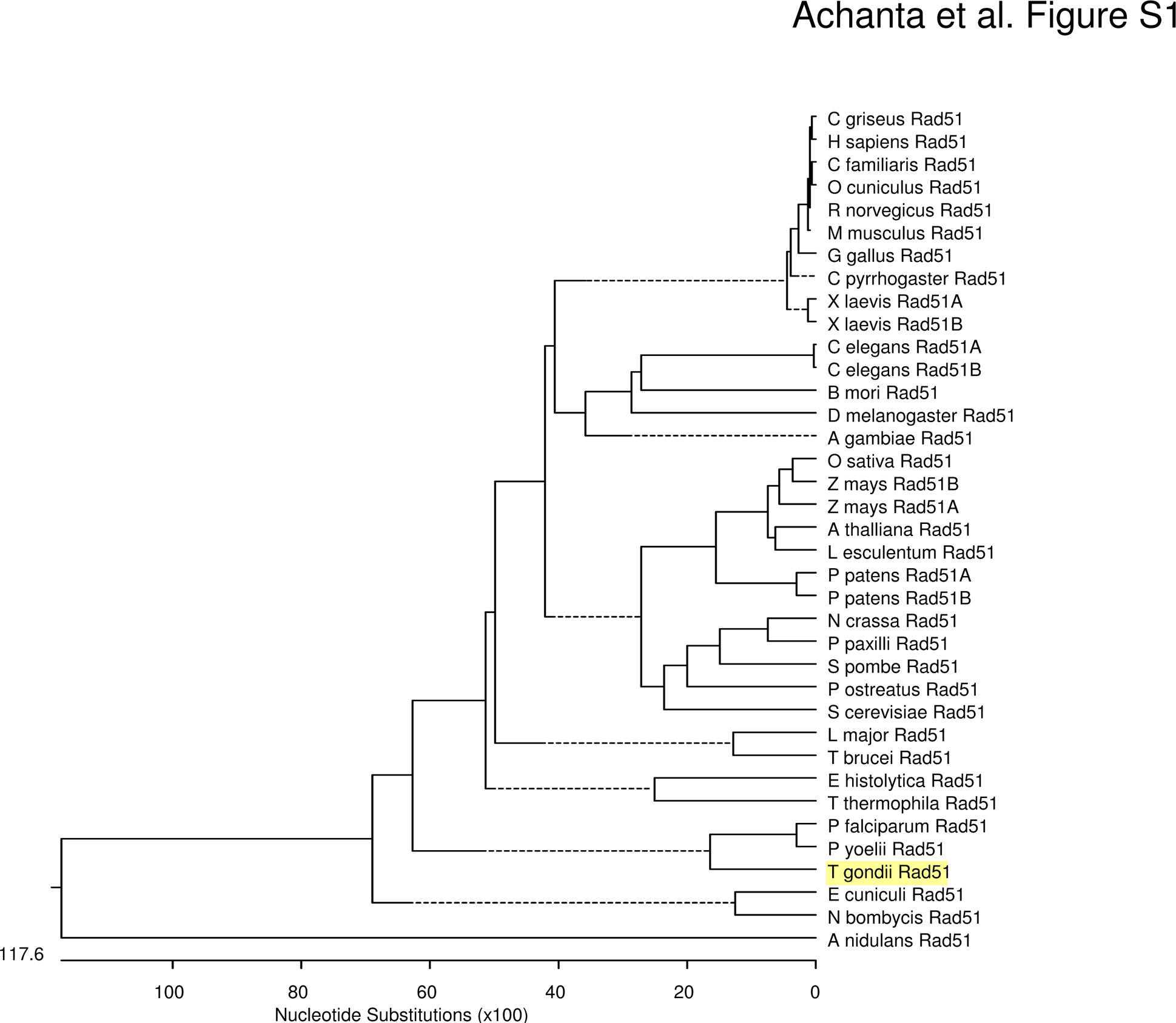

Supplement: Figure S1 — Phylogenetic analysis of eukaryotic Rad51 proteins using Clustal method (Meg align, DNA star). T. gondii Rad51 is highlighted. (TIF) [file pone.0041925.s001.tif]

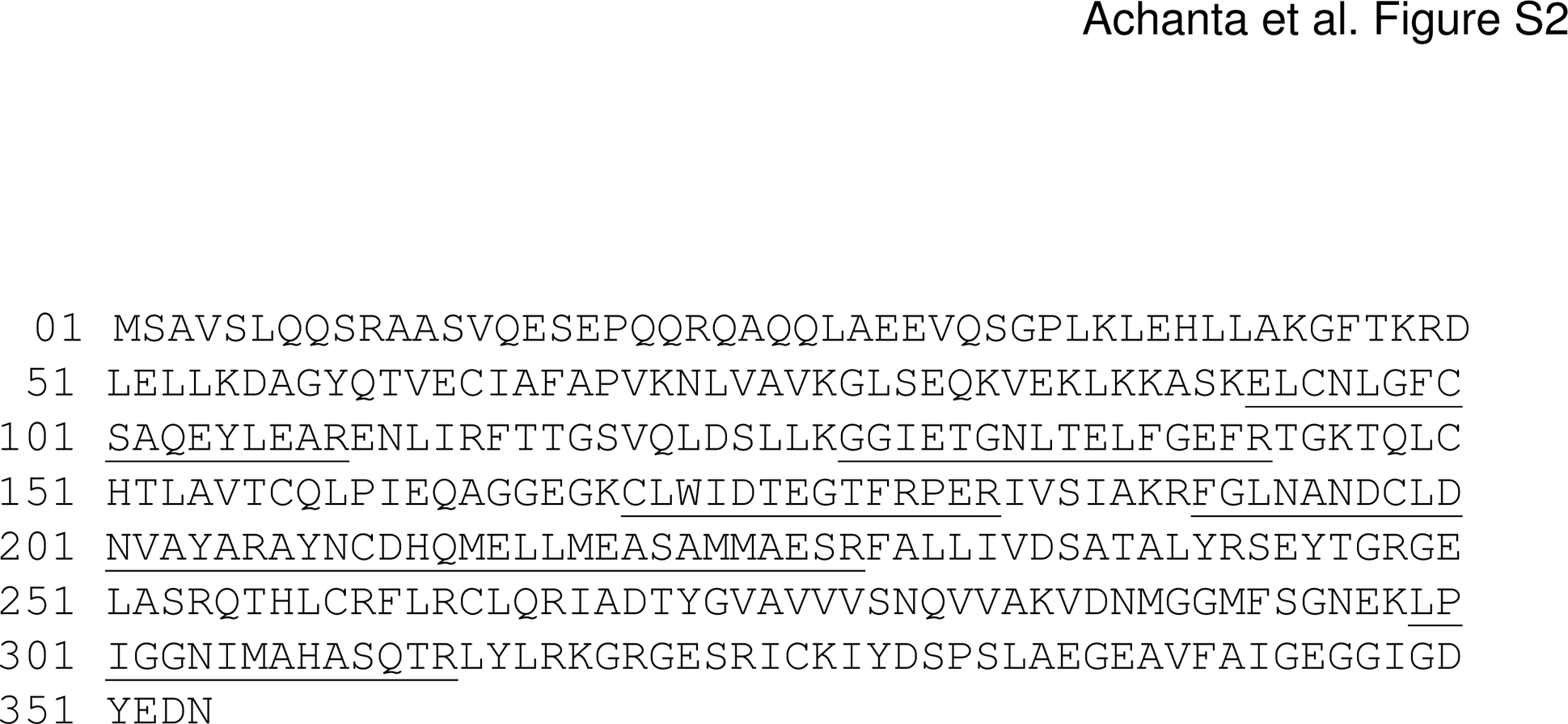

Supplement: Figure S2 — Amino acid sequence of TgRad51 protein from RH strain. The underlined peptide sequences were generated from MS-MS analysis. (TIF) [file pone.0041925.s002.tif]
